# Synthesis of NaYF_4_:Yb^3+^, Er^3+ ^upconversion nanoparticles in normal microemulsions

**DOI:** 10.1186/1556-276X-6-539

**Published:** 2011-10-03

**Authors:** Shu-Nan Shan, Xiu-Ying Wang, Neng-Qin Jia

**Affiliations:** 1 Department of Chemistry, Shanghai Normal University, Shanghai 200234, P.R. China

## Abstract

An interface-controlled reaction in normal microemulsions (water/ethanol/sodium oleate/oleic acid/*n*-hexane) was designed to prepare NaYF_4_:Yb^3+^, Er^3+ ^upconversion nanoparticles. The phase diagram of the system was first studied to obtain the appropriate oil-in-water microemulsions. Transmission electron microscopy and X-ray powder diffractometer measurements revealed that the as-prepared nanoparticles were spherical, monodisperse with a uniform size of 20 nm, and of cubic phase with good crystallinity. Furthermore, these nanoparticles have good dispersibility in nonpolar organic solvents and exhibit visible upconversion luminescence of orange color under continuous excitation at 980 nm. Then, a thermal treatment for the products was found to enhance the luminescence intensity. In addition, because of its inherent merit in high yielding and being economical, this synthetic method could be utilized for preparation of the UCNPs on a large scale.

## Introduction

The synthesis and spectroscopy of NaYF_4_:Yb^3+^, Er^3+ ^upconversion nanoparticles (UCNPs) have attracted a tremendous amount of attention because of their potential use in bioanalysis and medical imaging recently [1-5]. Upconversion was first recognized and formulated by Auzel in the mid-1960s [6], which is a process where low energy light, usually near-infrared (NIR) or infrared (IR), is converted to higher energies, ultraviolet (UV) or visible, via multiple absorptions or energy transfers. Up to now, several synthetic paths have been reported to obtain UCNPs, such as co-precipitation [2], hydrothermal, or solvothermal processing [7-11], liquid-solid two-phase approach [12], co-thermolysis of trifluoroacetate [13-17], decomposition of carbonate [18], diffusion-limited growth [19], and ionic liquid-assisted technique [20].

It is known that an important prerequisite for the applications of UCNPs is the availability of small and monodisperse nanoparticles [1]. Recently, the synthesis of various inorganic nanoparticles in normal microemulsions attracts our attention [21]. In the normal microemulsions, reactions are taking place at the interface of the normal micelles. Owing to the polarity inverse caused by the neutralization, the particles can be transferred from water phase to the oil phase. However, to the best of our knowledge, there is no study about the synthesis of NaYF_4_:Yb^3+^, Er^3+ ^UCNPs by this method. Therefore, we designed an oil/water interface-controlled reaction in normal microemulsions (water/surfactant/*n*-hexane) to produce NaYF_4_:Yb^3+^, Er^3+ ^UCNPs. The products are small, monodisperse, and high-yielding. They show good dispersibility in nonpolar organic solvents and emit visible upconversion luminescence under 980 nm excitation. Moreover, this synthetic strategy is very facile and less costly, which could be applied to mass-production.

## Results and discussion

**Figure 1:**
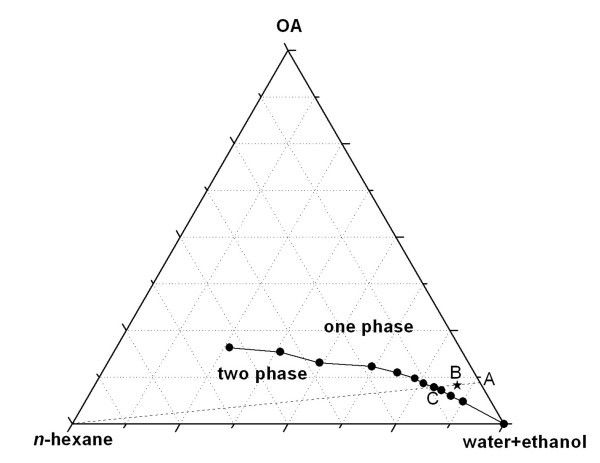
**Empirical phase diagram of the water/ethanol/NaOA/OA/*n*-hexane microemulsions**.

First, the phase behavior of the system was studied to obtain the appropriate microemulsions. Figure 1 shows the empirical phase diagram of the water/ethanol/sodium oleate (NaOA)/oleic acid (OA)/*n*-hexane mixtures at 298 K. Because of the complexity of the five-component system, the phase diagram was simplified to a ternary phase diagram, which is composed of total OA (including the part to generate NaOA with sodium hydroxide), water plus ethanol, and *n*-hexane. The composition is described using volume fractions. The water/ethanol ratio is always 1:1. The NaOA/OA molar ratio is always 2:3, and the total volume of OA is considered as the surfactant volume. The phase diagram is determined by gradual addition of *n*-hexane to a one-phase water/ethanol/NaOA/OA mixture with a constant volume fraction. For example, we begin from point A, and reach a critical point C where the solution starts showing a two-phase character.

The result shows that the one-phase/two-phase envelope extends from the point at 100% water plus ethanol to the point at 26.23% water plus ethanol, 20.45% OA, and 53.32% *n*-hexane, and the two-phase part is located in the lower OA region. Obviously, with an increase of the ratio of OA/(water plus ethanol), more *n*-hexane can be dissolved into their mixtures to form a stable system. The actual point (point B) we used is located in the right-bottom region, where the oil-in-water microemulsions are formed.

**Figure 2:**
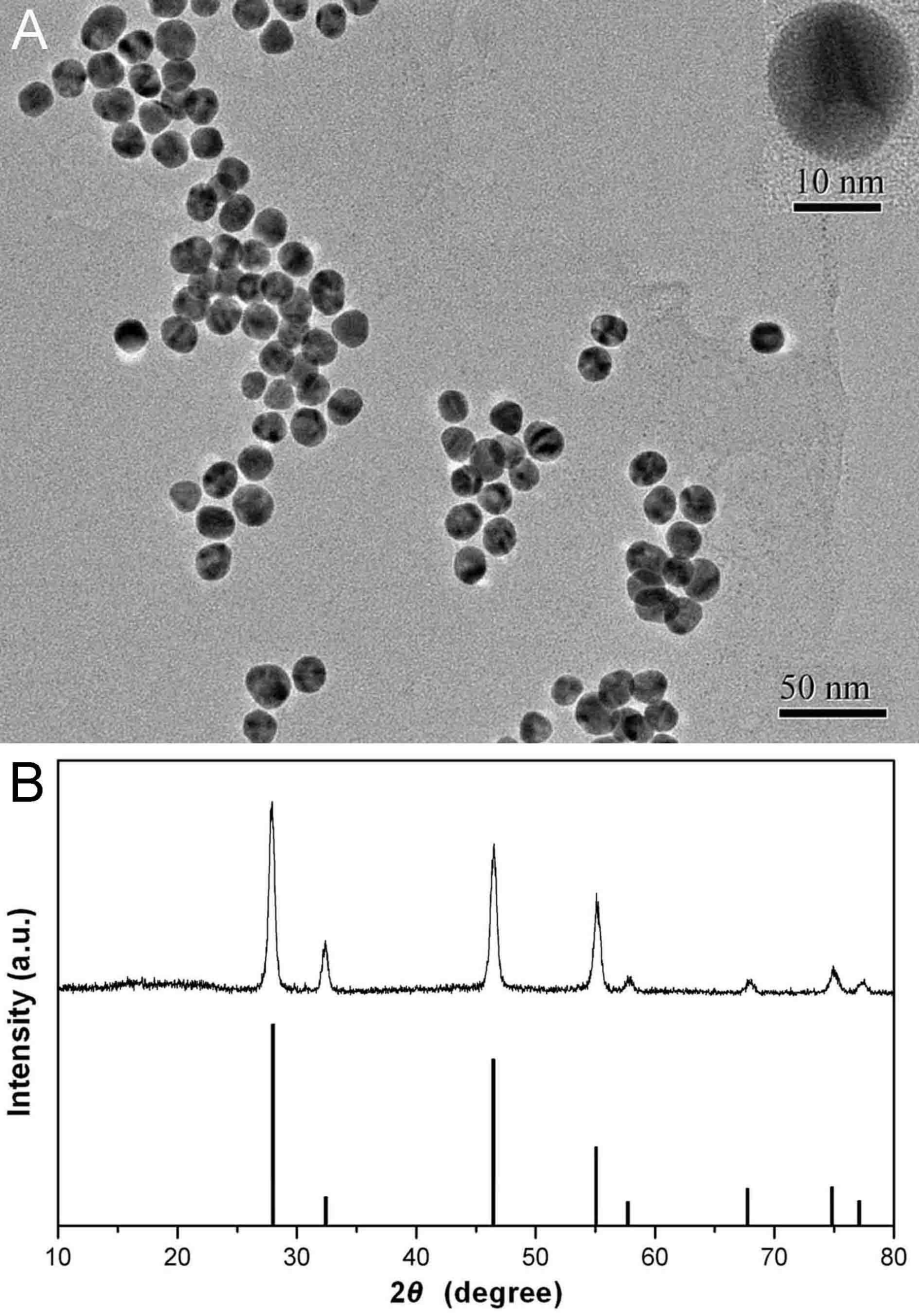
**Characterization data for NaYF_4_: 20% Yb^3+^, 2% Er^3+ ^UCNPs**. **(A) **TEM image (Inlet: HRTEM image of a single nanocrystal). **(B) **XRD pattern of the sample and the calculated line pattern for cubic phase of NaYF_4 _(JCPDS card, No. 77-2042).

Figure 2 shows the characterization data for the NaYF_4_:20% Yb^3+^, 2% Er^3+ ^sample. The TEM image (Figure 2A) demonstrates that the synthesized particles are roughly spherical, monodisperse with the size uniformity of about 20 nm in diameter. The X-ray powder diffractometer (XRD) pattern (Figure 2B) shows well-defined peaks, indicating the high crystallinity of the synthesized material, and the peak positions and intensities from the experimental XRD pattern match closely with the calculated pattern for cubic phase of NaYF_4 _(JCPDS card, No. 77-2042). From the line broadening of the diffraction peaks, the crystallite size of the sample was determined to be approximately 18 nm using the Debye-Scherrer formula, which corresponds to the particle size determined from the TEM result.

**Figure 3:**
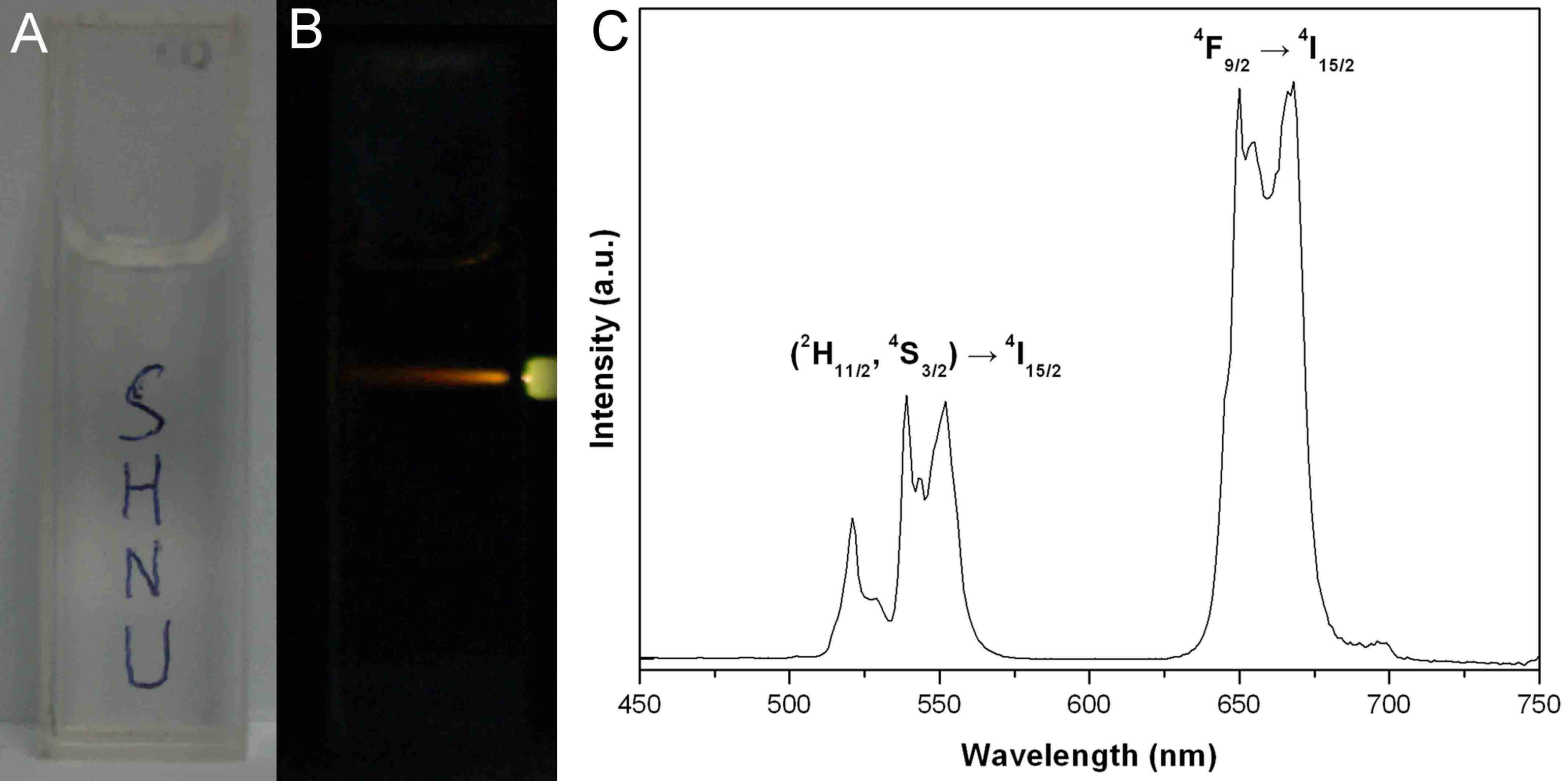
**Colloidal solutions of NaYF_4_:20% Yb^3+^, 2% Er^3+ ^sample in *n*-hexane**. **(A) **The solution showing its transparency. **(B) **Visible upconversion luminescence excited by 980 nm laser oxide. **(C) **Upconversion luminescence emission spectrum.

The NaYF_4_:Yb^3+^, Er^3+ ^UCNPs can easily be dispersed in nonpolar solvents (such as *n*-hexane, cyclohexane) to form homogenous colloidal solutions. Figure 3A shows images of a 1 wt.% solution of NaYF_4_:20% Yb^3+^, 2% Er^3+ ^UCNPs in *n*-hexane, demonstrating its transparency. The visible upconversion luminescence can be observed when the solution is excited at 980 nm with a power density of 1.2 kW/cm^2 ^(Figure 3B). The corresponding upconversion luminescence spectrum is also shown in Figure 3C. There are three major emission bands at 520-530 nm (green light), 540-550 nm (green light), and 650-670 nm (red light), which are assigned to the ^2^H_11/2 _to ^4^I_15/2_, ^4^S_3/2 _to ^4^I_15/2_, and ^4^F_9/2 _to ^4^I_15/2 _transitions of Er^3+ ^ion, respectively. Under 980 nm excitation, the absorption of the first photon can elevate Yb^3+ ^ion to the ^2^F_5/2 _level from ground state, and then it can transfer the energy to the Er^3+ ^ion. This energy transfer can promote Er^3+ ^ion from ^4^I_15/2 _level to the ^4^I_11/2 _level and from the ^4^I_11/2 _level to the ^4^F_7/2 _by another energy transfer upconversion process (or a second 980 nm photon) if the ^4^I_11/2 _level is already populated. Then, the Er^3+ ^ion can relax nonradiatively to the ^2^H_11/2 _and ^4^S_3/2 _levels, and the green emissions occur (^2^H_11/2 _→ ^4^I_15/2 _and ^4^S_3/2 _→ ^4^I_15/2_). Alternatively, the ion can further relax and populate the ^4^F_9/2 _level leading to the red emission (^4^F_9/2 _→ ^4^I_15/2_) [8,22]. The curve also shows that red emissions are much stronger than green emissions, so the products present light of orange color on the whole (Figure 3B).

**Figure 4:**
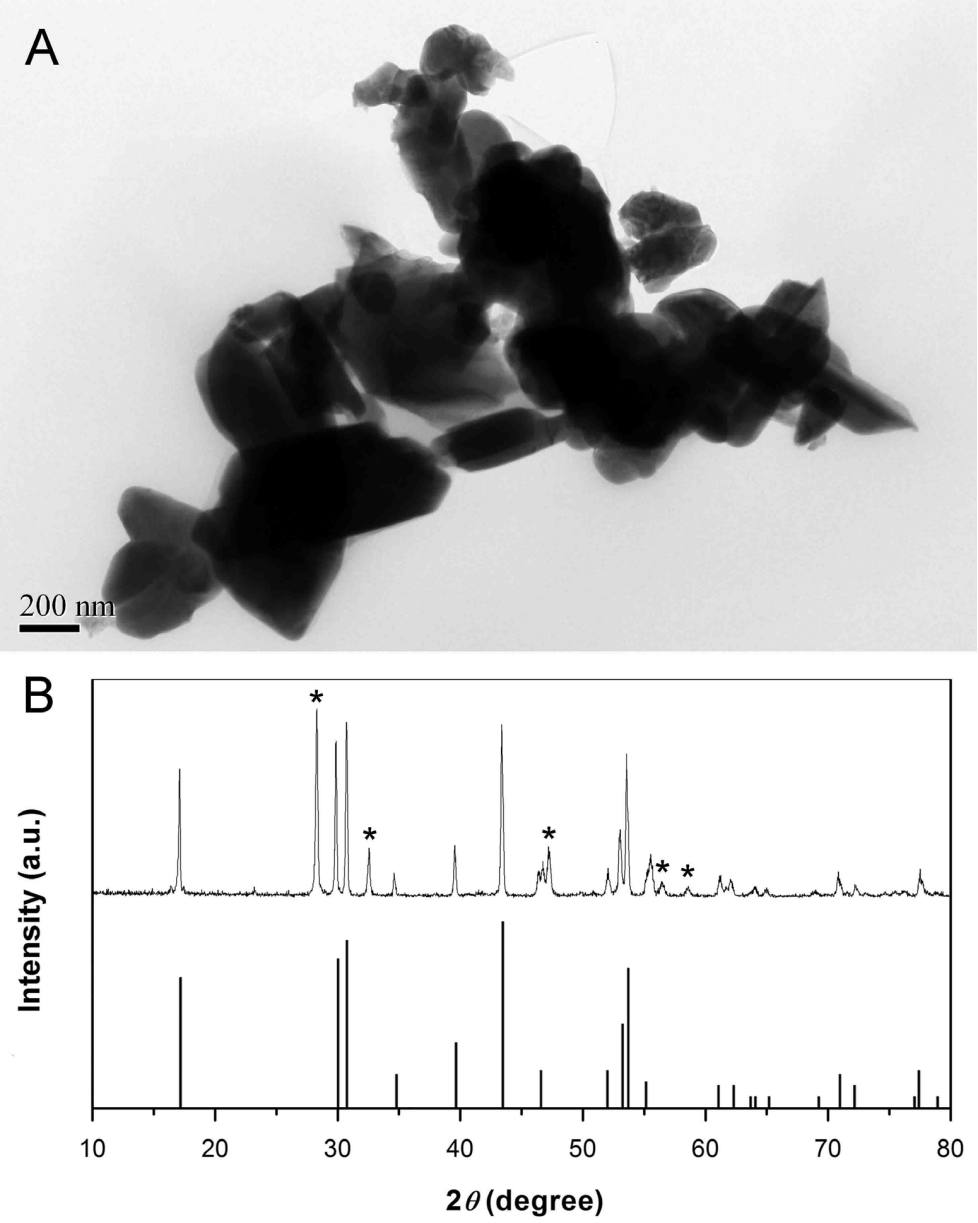
**Characterization data for NaYF_4_: 20% Yb^3+^, 2% Er^3+ ^UCNPs after annealing**. **(A) **TEM image. **(B) **XRD pattern (cubic phase is marked with asterisks) and the calculated line pattern for hexagonal phase of NaYF_4 _(JCPDS card, No. 28-1192).

**Figure 5:**
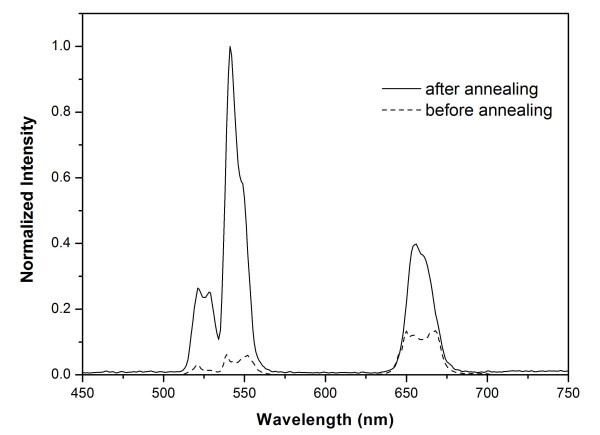
**Upconversion luminescence emission spectra of the nanoparticles before (dash line) and after (solid line) annealing**.

It is noted that the as-prepared nanoparticles are cubic phase, whose fluorescence efficiency is at least one-order of magnitude less than that of the hexagonal phase [8]. A thermal treatment at ca. 400-600°C was reported to transform the cubic phase to the hexagonal phase, but which led to undesirable particle growth and agglomeration [2]. We carried out the annealing of the as-prepared nanoparticles under N_2 _atmosphere by heating them to 600°C, and maintaining this temperature for 5 h. After annealing, the particles aggregated into larger clusters (Figure 4A), and the XRD pattern (Figure 4B) shows that hexagonal NaYF_4_:Yb^3+^, Er^3+ ^phase emerged in addition to the already existing cubic pattern (marked with asterisks), which implies that the particles transformed partially from cubic phase to hexagonal phase by annealing. In addition, upconversion luminescence emission spectrum (Figure 5) was obtained after ultrasonic dispersion of a 1 wt.% solution of the products in *n*-hexane, compared with the spectrum of nanoparticles before annealing, its green emission plays a dominant role, and the overall emissions are much stronger than those for cubic phase products.

## Conclusions

In summary, we designed a method of normal microemulsions to prepare NaYF_4_:Yb^3+^, Er^3+ ^UCNPs, which are small, monodisperse, and have good dispersibility in nonpolar organic solvents. Besides, the products exhibited visible upconversion luminescence under 980 nm excitation and a thermal treatment was proved to be able to strengthen the luminescence intensity. This method has its inherent merit in high yielding and being economical. Further study is currently underway to functionalize these synthesized UCNPs for their applications in biolabel and medical imaging.

## Materials and methods

All reagents used in this study, including sodium hydroxide, oleic acid, ethanol, *n*-hexane, sodium fluoride, and Ln(NO_3_)_3 _· 6H_2_O (Ln = Y, Yb, and Er, 99.99%) salt, were of analytical grade from Sinopharm Chemical Reagent Co., Ltd. (Shanghai, China). These chemicals were used without further purification. Water used in the experiment was double distilled.

In a typical synthetic route, sodium hydroxide (400 mg) was dissolved in a mixture of water (20 mL) and ethanol (30 mL), followed by the addition of oleic acid (7.4 mL) and *n*-hexane (4 mL); this formed a bright yellow transparent solution. Then, two separate aqueous solutions (5 mL) of Ln(NO_3_)_3 _(0.8 mmol, Y:Yb:Er = 78:20:2) and sodium fluoride (3.2 mmol) were added to the above microemulsions one after the other with vigorous stirring. Then, the solution was transferred to a Teflon-lined stainless steel autoclave and heated at 180°C for 6 h. When the autoclave was cooled down to room temperature, the products were found deposited at the bottom. Then, *n*-hexane (30 mL) was added to destroy the one-phase solution and form a two-phase mixture, so the hydrophobic colloidal NaYF_4_:20% Yb^3+^, 2% Er^3+ ^UCNPs were extracted into the upper layer (*n*-hexane region). With precipitation by additional ethanol, and highspeed centrifugation, the white products (yield: 85%) were re-dispersed in *n*-hexane to bring out a transparent colloidal solution.

The structure and morphology of NaYF_4_:20% Yb^3+^, 2% Er^3+ ^UCNPs were characterized by XRD and TEM. The obtained samples were characterized by XRD using a Brucker D8-advance X-ray diffractometer with Cu_Ka _radiation (λ = 1.5418 Å). The low- and high-resolution transmission electron microscopy (HRTEM) was performed on a JEOL JEM-3010 electron microscope operated at 300 kV. The upconversion emission spectra of NaYF_4_:20% Yb^3+^, 2% Er^3+ ^UCNPs were acquired using a Jobin-Yvon Fluorolog-3 fluorescence spectrometer system equipped with an external 0-1300 mW adjustable laser (980 nm, Beijing Hi-Tech Optoelectronic Co., China) as the excitation source, instead of the Xenon source in the spectrophotometer, and with an optic fiber accessory.

## Competing interests

The authors declare that they have no competing interests.

## Authors' contributions

SNS and XYW carried out the phase diagram studies. SNS participate in the sequence studies and drafted the manuscript. NQJ conceived of the study, and participated in its design and coordination and helped to draft and revise the manuscript.

## References

[B1] Van de Rijke F Zijlmans H Li S Vail T Rapp AK Niedbala RS Tanke HJ Up-converting phosphor reporters for nucleic acid microarrays Nat Biotechnol 2001 19 273 276 10.1038/85734 11231563

[B2] Yi GS Lu HC Zhao SY Ge Y Yang WJ Chen DP Guo LH Synthesis, characterization, and biological application of size-controlled nanocrystalline NaYF_4_:Yb, Er infrared-to-visible up-conversion phosphors Nano Lett 2004 4 2191 2196 10.1021/nl048680h

[B3] Kuningas K Rantanen T Ukonaho T Lövgren T Soukka T Homogeneous assay technology based on upconverting phosphors Anal Chem 2005 77 7348 7355 10.1021/ac0510944 16285685

[B4] Nyk M Kumar R Ohulchanskyy TY Bergey EJ Prasad P High contrast in vitro and in vivo photoluminescence bioimaging using near infrared to near infrared up-conversion in Tm^3+ ^and Yb^3+ ^doped fluoride nanophosphors Nano Lett 2008 8 3834 3838 10.1021/nl802223f 18928324 PMC3523349

[B5] Wang F Liu XG Recent advances in the chemistry of lanthanide-doped upconversion nanocrystals Chem Soc Rev 2009 38 976 989 10.1039/b809132n 19421576

[B6] Auzel F Upconversion and anti-stokes processes with f and d ions in solids Chem Rev 2004 104 139 173 10.1021/cr020357g 14719973

[B7] Wang LY Yan RX Huo ZY Wang L Zeng JH Bao J Wang X Peng Q Li YD Fluorescence resonant energy transfer biosensor based on upconversion-luminescent nanoparticles Angew Chem Int Ed 2005 44 6054 6057 10.1002/anie.200501907 16118828

[B8] Zeng JH Su J Li ZH Yan RX Li YD Synthesis and upconversion luminescence of hexagonal-phase NaYF_4_:Yb, Er^3+ ^phosphors of controlled size and morphology Adv Mater 2005 17 2119 2123 10.1002/adma.200402046

[B9] Zhang F Wan Y Yu T Zhang FQ Shi YF Xie SH Li YG Xu L Tu B Zhao DY Uniform nanostructured arrays of sodium rare-earth fluorides for highly efficient multicolor upconversion luminescence Angew Chem Int Ed 2007 46 7976 7979 10.1002/anie.200702519 17849413

[B10] Zhang F Li J Shan J Xu L Zhao DY Shape, size, and phase-controlled rare-earth fluoride nanocrystals with optical up-conversion properties Chem Eur J 2009 15 11010 11019 10.1002/chem.200900861 19739209

[B11] Wang X Zhuang J Peng Q Li YD A general strategy for nanocrystal synthesis Nature 2005 437 121 124 10.1038/nature03968 16136139

[B12] Wei Y Lu FQ Zhang XR Chen DP Synthesis of oil-dispersible hexagonal-phase and hexagonal-shaped NaYF_4_:Yb, Er nanoplates Chem Mater 2006 18 5733 5737 10.1021/cm0606171

[B13] Mai HX Zhang YW Si R Yan ZG Sun LD You LP Chun CH High-quality sodium rare-earth fluoride nanocrystals: controlled synthesis and optical properties J Am Chem Soc 2006 128 6426 6436 10.1021/ja060212h 16683808

[B14] Boyer JC Vetrone F Cuccia LA Capobianco JA Synthesis of colloidal upconverting NaYF_4 _nanocrystals doped with Er^3+^, Yb^3+ ^and Tm^3+^, Yb^3+ ^via thermal decomposition of lanthanide trifluoroacetate precursors J Am Chem Soc 2006 128 7444 7445 10.1021/ja061848b 16756290

[B15] Yi GS Chow GM Synthesis of hexagonal-phase NaYF_4_:Yb, Er and NaYF_4_:Yb, Tm nanocrystals with efficient up-conversion fluorescence Adv Funct Mater 2006 16 2324 2329 10.1002/adfm.200600053

[B16] Mishra S Daniele S Ledoux G Jeanneauc E Joubert MF Heterometallic Na-Y(Ln) trifluoroacetate diglyme complexes as novel single-source precursors for upconverting NaYF_4 _nanocrystals co-doped with Yb and Er/Tm ions Chem Commun 2010 46 3756 3758 10.1039/b921474g 20401365

[B17] Niu WB Wu SL Zhang SF Li L Synthesis of colour tunable lanthanide-ion doped NaYF_4 _upconversion nanoparticles by controlling temperature Chem Commun 2010 46 3908 3910 10.1039/c002615h 20414492

[B18] Schäfer H Ptacek P Eickmeier H Haase M Synthesis of hexagonal Yb^3+^, Er^3+^-doped NaYF_4 _nanocrystals at low temperature Adv Funct Mater 2009 19 3091 3097 10.1002/adfm.200900642

[B19] Li ZQ Zhang Y An efficient and user-friendly method for the synthesis of hexagonal-phase NaYF_4_:Yb, Er/Tm nanocrystals with controllable shape and upconversion fluorescence Nanotechnology 2008 19 345606/1 345606/16 10.1088/0957-4484/19/34/345606 21730655

[B20] Liu XM Zhao JW Sun YJ Song K Yu Y Du C Kong XG Zhang H Ionothermal synthesis of hexagonal-phase NaYF_4_:Yb^3+^, Er^3+^/Tm^3+ ^upconversion nanophosphors Chem Commun 2009 43 6628 6630 10.1039/b915517a 19865672

[B21] Ge JP Chen W Liu LP Li YD Formation of disperse nanoparticles at the oil/water interface in normal microemulsions Chem Eur J 2006 12 6552 6558 10.1002/chem.200600454 16773667

[B22] Boyer JC Cuccia LA Capobianco JA Synthesis of colloidal upconverting NaYF_4_:Er^3+^/Yb^3+ ^and Tm^3+^/Yb^3+ ^monodisperse nanocrystals Nano Lett 2007 7 847 852 10.1021/nl070235+ 17302461

